# Validation of the Chinese version of PedsQL™ brain tumor module

**DOI:** 10.3389/fped.2023.1277223

**Published:** 2023-10-30

**Authors:** Juan Wang, Jin Li, Xiaofan Jiang, Pei Sun, Xia Li, Guanyi Wang

**Affiliations:** ^1^ Department of Neurosurgery, Xijing Hospital, Airforce Military Medical University (Fourth Military Medical University), Xi’an, China; ^2^ Nursing Department of Zhengzhou People's Hospital, Zhengzhou, China; ^3^ Department of Neurosurgery, Xi’an Children’s Hospital, Xi’an, China

**Keywords:** pediatric brain tumors, health-related quality of life, PedsQL, children, translation

## Abstract

**Background:**

The study introduced the Pediatric Quality of Life Inventory™ (PedsQL™) brain tumor module for the first time in China. Further, the Chinese version of the PedsQL™ brain tumor module was developed and its feasibility, reliability, and validity were investigated.

**Methods:**

A total 129 cases completed the assessment. Feasibility was evaluated according to the percentage of missing items and the time required to complete the questionnaire. Internal consistency, retest reliability, and split-half reliability were tested to confirm reliability. We evaluated validity by testing content validity, construct validity, and criterion-related validity. The consistency between the child-self and parent-proxy reports was analyzed by calculating the correlation coefficient (r value) between them.

**Results:**

The Cronbach's alpha values for all subscales were above 0.7 and many subscales scored more than 0.9. The intra-class correlation coefficients of retest reliability were higher than 0.9. The split-half reliability scores for all subscales were higher than 0.6. The factor-item correlations ranged between 0.575–0.922 in the child report and 0.492–0.949 in the parent report. Exploratory factor analyses produced five factors corresponding to each subscale in the child report and six factors in the parent report.

**Conclusion:**

The feasibility, reliability, and validity of the Chinese PedsQL™ brain tumor module were ascertained through this study. This module can be used to effectively monitor children with brain tumors and conduct descriptive or exploratory studies to determine the risk factors affecting their quality of life. This would help develop a new basis for formulating measures to improve patient prognosis and quality of life.

## 1. Introduction

Brain tumors and other central nervous system (CNS) tumors have surpassed leukemia to become the most common cancers in children (1). With the development of neurosurgical technology, the five-year survival rate of pediatric brain tumor patients (PBTs) has increased to nearly 80% (2). The improvement in survival rate suggests that the quality of life of PBTs deserves more attention. Children receiving treatment usually experience symptoms such as pain, nausea, and fatigue. Many children may still experience issues with the nervous and endocrine systems even after treatment (3). In addition to physical problems, many PBTs may also have psychological problems, such as cognitive impairment and social disorder, resulting in a serious decline in their quality of life (4–6).

Health-related quality of life (HRQOL) has become an international research hotspot in the medical field. HRQOL is a multidimensional concept that includes physical, psychological, and social health (7). With the extensive development of HRQOL research, the research on quality of life gradually began to be applied to PBTs. To effectively evaluate the quality of life of patients with brain tumors, an efficient evaluation scale is necessary. Therefore, experts have developed some scales specifically used to evaluate HRQOL of patients with brain tumors, such as the head and neck cancer-specific scale of the Functional Assessment of Cancer Therapy (FACT) (8), the head and neck questionnaire (UWQOL) of the University of Washington (9), and the head and neck cancer-specific module of the European Cancer Research and Treatment Organization (EORTC QLQ-BN20) (10); however, these scales are not applicable to children with brain tumors.

The Pediatric Quality of Life Inventory ™ (PedsQL™) is a special quality of life measurement tool for children developed by Varni et al. (7). The inventory has good reliability and validity and has been used in many previous studies (11–14). Since its development in 1978, PedsQL™ has formed a complete modular evaluation system for children's quality of life. The scale consists of general core scales for measuring the common part of children's quality of life and disease-specific modules for measuring children's quality of life with different diseases. The PedsQL™ brain tumor module is one of the few effective tools to accurately evaluate HRQOL in children with brain tumors (15). Each set of scales was divided into four exclusive scales based on age: 2–4 years old, 5–7 years old, 8–12 years old, and 13–18 years old. While the 2–4 years old scale only includes parents' report, all other scales include both children's self-assessment report and parents' report (16). In 2006, Palmer et al. (15) first confirmed that the PedsQL™ brain tumor module has good reliability and validity in evaluating the quality of life of PBTs, and can effectively reflect the cognitive, neurological, endocrine system, and social and emotional problems of children (15). More than 10 countries including Japan and France have introduced the PedsQL™ brain tumor module and formed a variety of versions suitable for different cultures and language environments, with good applicability (17, 18). However, this module has not yet been introduced in China. The present study is the first to introduce the PedsQL™ brain tumor module in China, and has developed the Chinese version of the PedsQL™ brain tumor module and investigated its feasibility, reliability, and validity. Through this, we intended to provide a new method for the evaluation of the quality of life of children with brain tumors in China and lend a new basis for formulating measures to improve the prognosis and quality of life of children.

## 2. Method

### 2.1. Patients’ recruitment

Parents and their children with brain tumors, treated in the Department of Neurosurgery of Xijing Hospital from January 2010 to September 2020, were recruited for this study. This study was approved by the ethics committee of Xijing Hospital and the number is KY20202071-F-1.The inclusion criteria were as follows: (a) Children diagnosed with brain tumors; (b) Age range of 2–18 years; (c) Course of disease should be more than 1 month; (d) At least a parent of the children with brain tumor with basic Chinese listening and speaking ability, and with an ability of communicating with the researcher without obstacles; and (e) Children and their parents should have provided informed consent to participate in the study and should participate voluntarily. The exclusion criteria were as follows: (a) Children with other major diseases affecting their quality of life and (b) Children and/or parents who cannot correctly understand the content of the scale or fill in the scale correctly.

### 2.2. Translation process

**Figure 1:**
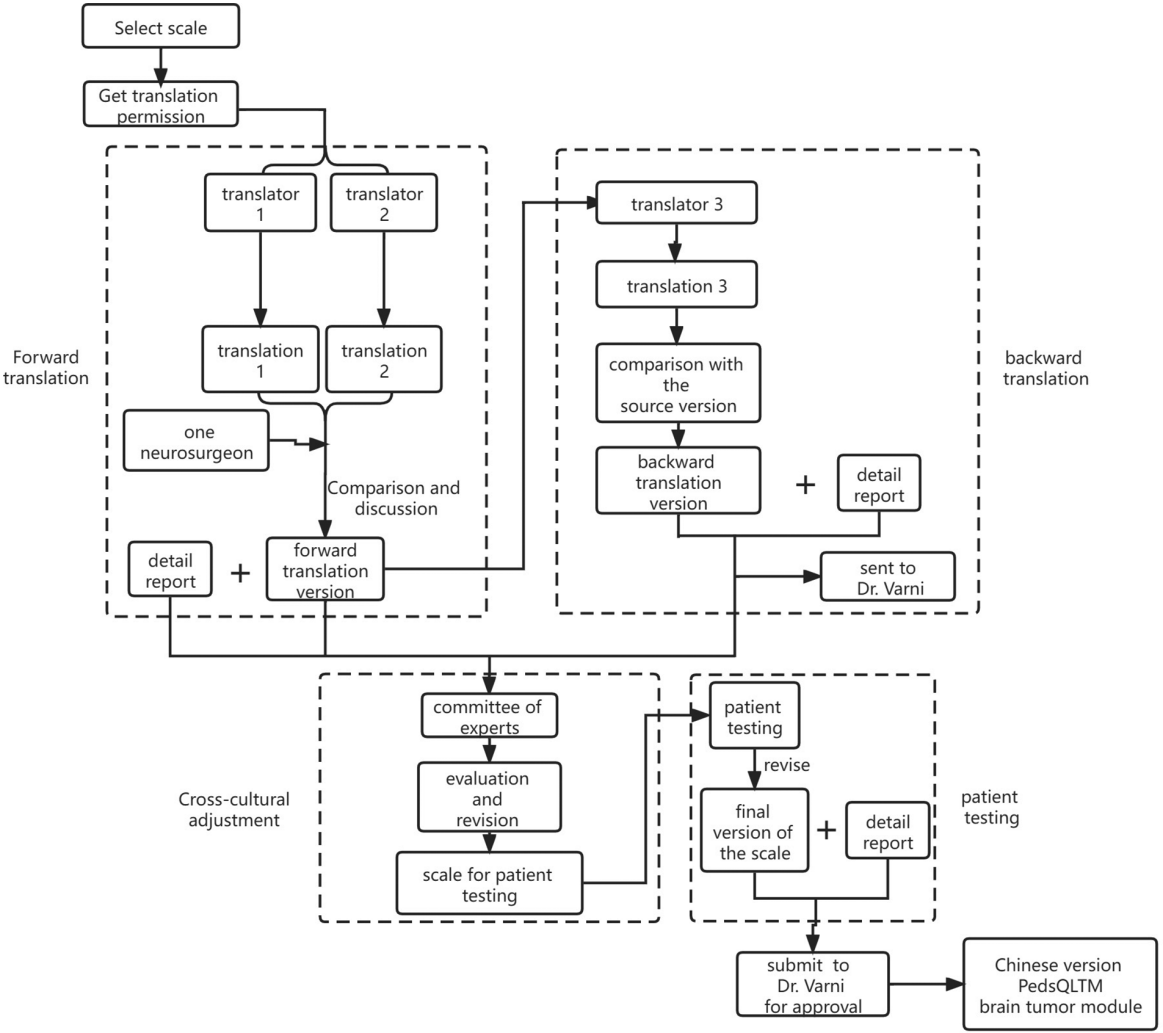
Flow chart of translation process.

After obtaining consent from the Mapi Research Trust and Dr. Varni, we began to translate the PedsQL™ brain tumor module (English version) into Chinese, following the Mapi Research Trust linguistic validation guidelines (19). A specific flowchart is shown in Figure 1.

The forward translation of this study was completed by two native Chinese translators, of whom one is a bilingualist with clinical professional knowledge and full understanding of the relevant contents of the scale and the other, a translator majoring in English. The entire process was completed by the two translators independently. After that, the two translators and a neurosurgeon jointly compared the two translations, discussed and refined the parts with obvious differences. Based on the principle of equivalent translation of the concept of the source scale and simplicity of the language after translation, they raised questions to Dr. Varni regarding disagreements, forming a single reconciled version.

The backward translation was completed by a professional translator whose mother tongue was English and the skilled language was Chinese. He translated the version obtained from forward translation back to English and without consulting the PedsQL™ brain tumor module in the whole process. After comparison with the source version and modification, the back-translated version was sent to Dr. Varni for obtaining his review before patient testing.

After performing cross-cultural adaptation of the scale, we started patient testing. Six children with brain tumors participated in patient testing, along with their parents: one each for 2–4 years old and 5–7 years old ranges and two each for 8–12 years old and 13–18 years old age ranges. During the cognitive interview, we found that children and their families generally had a good understanding of the content and expression of the questionnaire. The time for children to complete the questionnaire was 4–9 min and their parents needed 3–6 min. The final version of the Chinese adaptation of the PedsQL™ brain tumor module was formed after obtaining the results. All the documents above were sent back to Dr. Varni for a final crosscheck and consent.

### 2.3. PedsQL™ brain tumor module

The PedsQL™ brain tumor module consists of six modules and 24 items: cognitive problems (7 items), pain and hurt (3 items), movement and balance (3 items), procedural anxiety (3 items), nausea (5 items), and worry (3 items). The parent proxy-report for the 2–4 years old group did not include the cognitive problem module. All child and parent reports containing responses for the questions used in the 8–18 years old group were based on a 5-point Likert-scale (0 = never a problem; 1 = almost never a problem; 2 = sometimes a problem; 3 = often a problem; 4 = almost always a problem). To facilitate the understanding and cooperation of the 5–7 years old group, the scale was simplified to a 3-point Likert scale (0 = not at all a problem; 2 = sometimes a problem; 4 = a lot of a problem). Items were reverse-scored and linearly transformed to a 0–100 scale (0 = 100, 1 = 75, 2 = 50, 3 = 25, 4 = 0) as higher scores indicate better HRQOL. Scale scores were computed as the sum of the items divided by the number of items answered, and the summary score was calculated as the sum of all the 24 items divided by the number of items answered.

### 2.4. PedsQL 4.0 generic core scales

The PedsQL 4.0 Generic Core Scales contains four modules and 23 items: physical functioning (8 items), emotional functioning (5 items), social functioning (5 items), and school functioning (5 items). The format, response scale, and scoring method were identical to those of the PedsQL™ brain tumor module. The scale was translated into Chinese and has good reliability and validity (20).

### 2.5. Data collection and quality control

Two nurses with at least five years of pediatric neurosurgery clinical nursing experience and two research students performed this investigation. All interviewers were trained before the investigation in interviewing and administering questionnaires. This study was approved by the ethics committee of Xijing Hospital. The researchers explained the purpose, significance, and research process of this investigation to the children and their parents, and informed them that they had the right to fill in the questionnaire voluntarily and withdraw from the study freely. After obtaining informed consent, the researchers explained the filling method of the questionnaire and distributed the PedsQL™ brain tumor module and PedsQL™ 4.0 generic core scales. The children and their parents completed the questionnaire independently. Among children aged 5–7 or those unable to read or write, the questionnaires were administered by their parents or researcher. After that, two students reviewed all the questionnaires and supplemented the missing items through repeated interviews or telephone interviews.

### 2.6. Statistical analysis

The feasibility was evaluated according to the percentage of missing items and the time required to complete the questionnaire. Three methods were used to test the reliability: (1) Internal consistency: Cronbach's alpha was used to evaluate the internal consistency; alpha value of 0.7–0.8 indicates that the scale was reliable and alpha value of 0.8–0.9 indicates that the reliability of the scale was very good. (2) Retest reliability: Assuming that there is no change in the status of a group of patients in a short time, each object was measured twice with the same scale, and the two results were tested using the intra-class correlation coefficient (ICC). The ICC is a value between 0 and 1. The higher the consistency of the two measurement results, the closer the ICC is to 1, the higher the test-retest reliability, and the better the stability of the scale and therefore, the more reliable the results. We selected 18 children who were hospitalized for more than 1 week and their parents to measure the test-retest reliability. (3) Split-half reliability: The items in the scale were randomly divided into two halves to obtain two total scores. The correlation coefficient between the two total scores was recorded as R, and 2R/(1 + R) was the split half reliability of the scale. When this coefficient is greater than 0.6, the split half reliability is good (20). The supplementary test-retest reliability cannot measure the defects of objects that change over time.

We also used three methods to test the validity of the Chinese version of the PedsQL™ brain tumor module: (1) Content validity: The content validity of the scale was analyzed by calculating the Pearson correlation coefficient (r value) between the items and subscales of the scale. The r value is between 0 and 1, and the higher the r value, the better the content validity. (2) Construct validity: In this study, exploratory factor analysis was used to test the structural validity of the scale. First, the Kaiser-Meyer-Olkin (KMO) value was calculated to judge whether the scale is suitable for factor analysis. Then, we determined the number of factors, extracted the common factors by principal component analysis, carried out rotation transformation, and sought the best analysis effect. The cumulative variance contribution rate of the extracted common factors should be greater than 40% and each item has a high factor compliance (>0.4) on its common factors (15). (3) Criterion-related validity: The Chinese version of the PedsQL 4.0, generic core scales (2–18 years old) was used as the calibration standard to test the calibration correlation validity. The higher the correlation coefficient (r value) between them, the better the correlation degree of the effective standard and the r value is between 0 and 1 (21).

The consistency between the child-self and parent-proxy reports was analyzed by calculating the correlation coefficient (r value) between them. All analyses were performed using SPSS 23.0, and statistical significance was set at *p *< 0.05. Power analysis using the findings from the original English version demonstrated that the minimum requisite sample size was 85 subjects (15). Besides, the sample size should be more than 100 to meet the minimum sample size for exploratory factor analysis.

## 3. Results

### 3.1. Sample characteristics

A total of 129 patients and their parents were included in the study and completed the assessment. The children were 2–18 years old, with a median age of eight years. The course of the disease ranged from 1 month to 125 months, with a median time of 35 months. A total of 125 patients received surgery; 31, radiotherapy; 45, chemotherapy; and 3, no treatment. See Table 1 for further details.

**Table 1:** Clinical characteristics of the pediatric brain tumor patients.

	Number of respondents	% of total
Gender		
Male	72	55.8
Female	57	44.2
Place of residence		
City	78	60.5
vCountryside	51	39.5
Is the only one child in the family		
Yes	88	68.2
No	41	31.8
Cancer diagnosis		
Pituitary adenoma	5	3.9
Glioma	39	30.2
Craniopharyngioma	25	19.4
Meningioma	3	2.3
Germinoma	5	3.9
Ependymoma	13	10.1
Medulloblastoma	25	19.4
Others	14	10.9
Surgical treatment		
Not accepted	4	3.1
Accepted	125	96.9
Radiotherapy		
Not accepted	98	76
Accepted	31	24
Chemotherapy		
Not accepted	84	65.1
Accepted	45	34.9
Relationship of parent to child		
Father	58	45
Mother	71	55

### 3.2. Descriptive statistics and feasibility

The maximum value for all scales was 100, whereas the minimum value ranged from 0 to 25. The parent reports' scores were higher than the child reports' scores in all subscales (Table 2). The time for children to complete the questionnaire was 4–9 min and their parents needed 3–6 min.

**Table 2:** Scale descriptives for Chinese version of PedsQL™ brain tumor module child and parent reports.

	Number (n)	Minimum	Maximum	Mean	SD
Child report					
Cognitive problems	106	25	100	69.29	16.97
Pain and hurt	106	0	100	73.51	21.89
Movement and balance	106	0	100	75.16	26.84
Procedural anxiety	106	0	100	45.68	31.77
Nausea	106	20	100	72.17	22.11
Worry	106	0	100	43.00	28.44
Parent report					
Cognitive problems	106	16.67	100	72.18	18.51
Pain and hurt	129	8.33	100	75.78	19.34
Movement and balance	129	8.33	100	78.49	24.11
Procedural anxiety	129	0	100	60.53	26.26
Nausea	129	20	100	73.37	21.86
Worry	129	0	100	59.04	26.02

### 3.3. Reliability

Internal consistency and retest reliability are shown in Table 3. The Cronbach's coefficient alpha values for all subscales were above 0.7 and many subscales scored more than 0.9, proving that the Chinese version of the PedsQL™ brain tumor module has good internal consistency. We chose 18 children, who were hospitalized for more than one week, and their parents to measure the retest reliability. As shown in Table 3, all the ICCs were higher than 0.9, indicating that all the subscales had high agreement in this test. For split-half reliability, all subscale scores were higher than 0.6 (Table 4).

**Table 3:** Internal consistency and retest reliability of Chinese version of PedsQL™ brain tumor module child and parent reports.

	Cronbach's alpha				Retest reliability
	2–4 years (*n* = 23)	5–7 years (*n* = 35)	8–12 years (*n* = 37)	13–18 years (*n* = 34)	ICC (*n* = 18)
Child report					
Cognitive Problems	–	0.861	0.929	0.800	0.989
Pain and Hurt	–	0.739	0.791	0.815	0.983
Movement and balance	–	0.742	0.777	0.727	0.990
Procedural Anxiety	–	0.928	0.986	0.977	0.985
Nausea	–	0.822	0.880	0.820	0.907
Worry	–	0.805	0.950	0.980	0.967
Parent report					
Cognitive problems	–	0.909	0.903	0.879	0.976
Pain and Hurt	0.773	0.791	0.755	0.818	0.964
Movement and balance	0.839	0.744	0.721	0.758	0.972
Procedural Anxiety	0.976	0.975	0.968	0.975	0.973
Nausea	0.798	0.882	0.884	0.791	0.956
Worry	0.973	0.924	0.940	0.976	0.965

**Table 4:** Split-half reliability of Chinese version of pedsQL™ brain tumor module child and parent reports.

	Split-half reliability			
	2–4 years (*n* = 23)	5–7 years (*n* = 35)	8–12 years (*n* = 37)	13–18 years (*n* = 34)
Child report				
Cognitive problems	–	0.920	0.906	0.716
Pain and hurt	–	0.626	0.685	0.780
Movement and balance	–	0.601	0.712	0.763
Procedural anxiety	–	0.817	0.886	0.874
Nausea	–	0.857	0.936	0.806
Worry	–	0.662	0.781	0.852
Parent report				
Cognitive problems	–	0.888	0.884	0.819
Pain and hurt	0.679	0.635	0.642	0.697
Movement and balance	0.646	0.653	0.812	0.721
Procedural anxiety	0.853	0.881	0.852	0.857
Nausea	0.723	0.902	0.907	0.812
Worry	0.843	0.776	0.723	0.894

### 3.4. Validity

As shown in Table 5, there is a certain correlation between each item and each subscale. Except for items 8 and 13 in the children's report and item 13 in the parents' report, the other correlation coefficients are all greater than 0.6, indicating that the Chinese version of the PedsQL™ brain tumor module has good content validity. The results of the factor analysis for the Chinese version of the PedsQL™ brain tumor module to test the construct validity are presented in Tables 6, 7. Exploratory factor analyses produced five factors corresponding to each subscale in the child report and six factors in the parent report. The factor-item correlations were between 0.575 and 0.922 in the child report and between 0.492 and 0.949 in the parent report. We used the Chinese version of the PedsQL™ 4.0 generic core scale as a calibration standard to test the calibration correlation validity of the Chinese version of the PedsQL™ brain tumor module. There was a high degree of consistency between the total scores of the two scales. There was good consistency between all the subscales and the total score of the calibration scale (Table 8).

**Table 5:** Content validity of Chinese version of PedsQL™ brain tumor module child and parent reports.

	Child report						Parent report					
	Cognitive Problem	Pain And hurt	Movement and balance	Procedure Anxiety	Nausea	Worry	Cognitive Problem	Pain And hurt	Movement and balance	Procedure Anxiety	Nausea	Worry
Cognitive Problem												
Item1	**.** **664**	.069	.062	.345	.551	.319	**.** **806**	.395	.181	.298	.314	.230
Item2	**.** **767**	.249	.262	.232	.392	.094	**.** **836**	.410	.325	.211	.280	.083
Item3	**.** **776**	.233	.169	.317	.476	.181	**.** **851**	.349	.199	.344	.417	.202
Item4	**.** **709**	.231	.152	.356	.468	.281	**.** **679**	.218	.088	.201	.411	.162
Item5	**.** **635**	.318	.335	.257	.347	.004	**.** **773**	.423	.319	.200	.413	.106
Item6	**.** **777**	.335	.286	.338	.540	.085	**.** **725**	.397	.267	.202	.456	.251
Item7	**.** **718**	.361	.300	.096	.460	.085	**.** **806**	.626	.311	.251	.449	.305
Pain and hurt												
Item8	.042	**.** **311**	.141	.139	.078	.075	.517	**.** **876**	.474	.144	.285	.143
Item9	.368	**.** **817**	.503	.187	.424	.106	.495	**.** **876**	.407	.099	.342	.068
Item10	.303	**.** **825**	.649	.346	.218	.031	.276	**.** **747**	.452	.053	.306	.133
Movement and balance												
Item11	.188	.589	**.** **909**	.161	.178	.087	.137	.423	**.** **912**	.044	.177	.018
Item12	.175	.656	**.** **950**	.214	.124	.079	.155	.441	**.** **941**	.023	.205	.015
Item13	.490	.379	**.** **462**	.297	.098	.053	.542	.427	**.** **419**	.221	.160	.131
Procedure Anxiety												
Item14	.384	.291	.221	**.** **970**	.435	.467	.304	.087	.031	**.** **970**	.502	.540
Item15	.362	.375	.282	**.** **988**	.434	.428	.299	.132	.027	**.** **978**	.509	.612
Item16	.372	.359	.265	**.** **987**	.436	.434	.311	.126	.115	**.** **968**	.539	.612
Nausea												
Item17	.491	.379	.266	.430	**.** **831**	.310	.402	.391	.336	.373	**.** **822**	.525
Item18	.478	.086	.062	.395	**.** **831**	.368	.357	.156	.046	.572	**.** **862**	.601
Item19	.491	.373	.336	.235	**.** **639**	.064	.482	.426	.453	.217	**.** **601**	.226
Item20	.648	.377	.186	.251	**.** **772**	.045	.477	.377	.128	.375	**.** **746**	.270
Item21	.454	.327	.090	.381	**.** **887**	.179	.328	.259	.105	.480	**.** **902**	.545
Worry												
Item22	.199	.081	.097	.422	.295	**.** **970**	.268	.174	.056	.613	.574	**.** **965**
Item23	.210	.100	.111	.470	.308	**.** **969**	.220	.172	.110	.582	.540	**.** **973**
Item24	.067	.038	.055	.403	.110	**.** **933**	.194	.055	.008	.529	.539	**.** **918**

Bold data represents the correlation coefficient between the item and the modules in which it is located.

**Table 6:** Construct validity of Chinese version of PedsQL™ brain tumor module child report.

**Child report** ***n* = 106, cumulative variance 76.021%**					
Cognitive problems					
It is hard for me to figure out what to do when something bothers me	**.** **604**	−.097	.321	.370	.199
I have trouble solving math problems	**.** **752**	.087	.131	.136	.089
I have trouble writing school papers or reports	**.** **764**	−.019	.203	.198	.169
It is hard for me to pay attention to things	**.** **637**	.076	.315	.273	.178
It is hard for me to remember what I read	**.** **624**	.263	−.003	.126	.207
It is hard for me to learn new things	**.** **691**	.163	.048	.332	.226
I get mixed up easily	**.** **695**	.226	−.076	.332	−.033
Pain and hurt					
I ache or hurt in my joints and/or muscles	**.** **677**	.478	.063	.105	.074
I hurt a lot	.440	**.** **603**	.125	.253	−.015
I get headaches	.261	**.** **717**	−.082	.097	.353
Movement and balance					
It is hard for me to keep my balance	.107	**.** **896**	.113	.069	.039
It is hard for me to use my legs	.129	**.** **914**	.087	−.022	.123
It is hard for me to use my hands	**.** **724**	.197	.066	−.258	.275
Procedural anxiety					
Needlesticks (i.e., injections, blood tests, IVs) hurt me	.247	.096	.311	.200	**.** **858**
I get scared when I have to have blood tests	.212	.184	.268	.216	**.** **876**
I get scared about needlesticks (i.e., injections, blood tests, IVs)	.223	.162	.276	.212	**.** **874**
Nausea					
I become sick to my stomach when I have medical treatments	.267	.260	.228	**.** **736**	.220
Food does not taste very good to me	.237	−.097	.308	**.** **774**	.193
I become sick to my stomach when I think about medical treatments	**.** **575**	.306	.094	.271	.063
I feel too sick to my stomach to eat	**.** **592**	.160	−.106	.607	.089
Some foods and smells make me sick to my stomach	.247	.128	.095	**.** **849**	.203
Worry					
I worry about side effects from medical treatments	.129	.079	**.** **922**	.182	.181
I worry about whether my medical treatments are working	.131	.092	**.** **907**	.180	.236
I worry that my cancer will come back or relapse	.092	.068	**.** **911**	.035	.232

Method: Principal component analysis with promax rotation. KMO = 0.847. Bold values indicate the largest factor loadings for each item.

**Table 7:** Construct validity of Chinese version of PedsQL™ brain tumor module parent report.

**Parent report** ***n* = 129, cumulative variance 77.379%**						
Cognitive problems						
Difficulty figuring out what to do when something bothers him/her	.524	**.** **598**	.144	.179	−.043	−.087
Trouble solving math problems	.548	**.** **626**	−.062	.170	−.038	.079
Trouble writing school papers or reports	.315	**.** **782**	.020	.235	.136	.015
Difficulty paying attention to things	.005	**.** **782**	.065	.011	.281	.015
Difficulty remembering what he/she reads	**.** **614**	.494	−.042	.109	.175	−.007
Difficulty learning new things	.164	**.** **726**	.183	−.048	.255	.225
Difficulty learning new things	**.** **689**	.459	.204	.029	.176	.079
Pain and hurt						
Aches in joints and/or muscles	**.** **828**	.052	.075	−.008	.121	.268
Having a lot of pain	**.** **809**	.028	−.012	−.065	.275	.219
Getting headaches	.265	.099	.103	−.106	.266	**.** **512**
Movement and balance						
Difficulty keeping his/her balance	.085	.039	−.012	−.009	.005	.949
Difficulty using his/her legs	.152	.025	−.027	.013	.018	**.** **944**
Difficulty using his/her hands	**.** **718**	.240	.047	.203	−.177	.022
Procedural anxiety						
Needlesticks (i.e., injections, blood tests, and IVs) causing him/her pain	.051	.130	.247	**.** **881**	.230	−.079
Getting anxious about having blood drawn	.122	.078	.340	**.** **873**	.183	−.040
Getting anxious about having needlesticks (i.e., injections, blood tests, IVs)	.091	.106	.346	**.** **877**	.177	.043
Nausea						
Becoming nauseated during medical treatments	.170	.204	.427	.082	**.** **597**	.308
Food not tasting very good to him/her	.005	.189	.404	.364	**.** **653**	−.011
Becoming nauseated while thinking about medical treatments	**.** **492**	.159	.076	.087	.412	.302
Feeling too nauseous to eat	.220	.277	.000	.203	**.** **745**	.029
Some foods and smells making him/her nauseous	.047	.127	.346	.246	**.** **790**	.062
Worry						
Worrying about side effects from medical treatments	.080	.097	**.** **885**	.314	.175	.016
Worrying about whether his/her medical treatments are working	.103	.025	**.** **911**	.284	.138	.077
Worrying that the cancer will reoccur or relapse	−.008	.079	**.** **847**	.242	.209	−.058

Method: Principal component analysis with promax rotation. KMO = 0.769. Bold values indicate the largest factor loadings for each item.

**Table 8:** Criterion-related validity of Chinese version of PedsQL™ brain tumor module child and parent reports.

	PedsQL 4.0 generic core scales	
PedsQL™ brain tumor module	Child report	Parent report
Cognitive problem	.710	.865
Pain and hurt	.668	.634
Movement and balance	.662	.650
Procedural anxiety	.605	.609
Nausea	.640	.641
Worry	.504	.528
Total	.857	.869

### 3.5. Consistency between child-self report and parent-proxy report

As shown in Table 9, ICCs between the same subscales of child-self reports and parent-proxy reports were all higher than 0.6. The ICCs of the total scores were as high as 0.89.

**Table 9:** The consistency between Chinese version of PedsQL™ brain tumor module child and parent reports.

Child report	Parent report						
	CP	PH	MB	PA	N	W	Total
Cognitive Problems (CP)	**0.86**	0.45	0.44	0.41	0.57	0.32	0.69
Pain and Hurt (PH)	0.65	**0.83**	0.55	0.20	0.38	0.05	0.67
Movement and Balance (MB)	0.49	0.67	**0.85**	0.10	0.42	0.14	0.54
Procedural Anxiety (PA)	0.29	0.17	0.20	**0.60**	0.37	0.31	0.44
Nausea (N)	0.55	0.56	0.30	0.50	**0.89**	0.47	0.64
Worry (W)	0.23	0.16	0.06	0.57	0.54	**0.67**	0.32
Total	0.64	0.55	0.58	0.38	0.70	0.43	**0.89**

Bold data represents the correlation coefficient between the same modules reported by parents and children.

## 4. Discussion

Currently, increasing attention is being paid to research on HRQOL of patients worldwide, and an increasing number of scales have been introduced in China to specifically study the HRQOL of children with certain diseases (21–24). However, there are no measurement tools that are be specifically suitable for PBTs in China. Our study is the first to develop the Chinese version of the PedsQL™ brain tumor module and demonstrate its feasibility, reliability, and validity in PBTs. Through the present study, we proved that this questionnaire can effectively reflect the HRQOL of PBTs.

We completed the translation according to the MAPI Research Trust linguistic validation guidelines. Adhering to the principles of conceptual equivalence, semantic equivalence, idiom equivalence, and experience equivalence, we added cultural adaptation following backward translation. After the patient testing, to ensure that the PBTs and their parents could easily and correctly understand the questionnaire, the Chinese version of the PedsQL™ brain tumor module was officially developed. The children who could not complete the questionnaire independently were helped by the investigator. Due to our strict inclusion criteria and the fact that the researchers helped the patients, no item was missed in both the child and parent reports. The minimal missing item responses indicated that PBTs and their parents could provide good quality data on the patients' HRQOL through the questionnaire. During patient testing, only a short time was required for the patients and their parents to complete the questionnaire. These results suggest that the Chinese version of the PedsQL™ brain tumor module has good feasibility and is convenient for use in the fast-paced work of outpatient clinics.

For all the scales for both child and parent reports, the Cronbach's alpha approached or exceeded the standard of 0.70, thus indicating its reliability. Most of the subscales exceeded an alpha of 0.90, indicating that this scale could be used for individual analysis and comparative analysis of HRQOL between the groups in clinical trials (25). Moreover, all the subscales in both the child and parent reports showed sufficient retest reliability and split-half reliability. These results show that the Chinese version of the PedsQL™ brain tumor module has good reliability.

We have completed enough tests to prove the scale's validity, such as content, construct, and criterion-related validity. The present study is the first to ascertain the scale's content validity by calculating the Pearson correlation coefficient (r value) between the items and subscales, which had not been confirmed in the original or other versions of the PedsQL™ brain tumor module. Except for three items, the correlation coefficients between all the other items and their subscales were greater than 0.6, indicating that the scale has good content validity in both child and parent reports. As expected, the Chinese version of the PedsQL™ brain tumor module performs well in terms of construct validity and calibration correlation validity, which is completely consistent with previous studies (15, 17, 18).

In our study, we observed that the scores of procedural anxiety and worry subscales were relatively lower than those of other subscales, which was consistent with the findings of Caru et al. (17) and Palmer et al. (15). This may be due to the severity of symptoms and treatment of PBTs (26), or trying to avoid hospital visits (27). Current studies have different opinions on the consistency of parents' and children's reports (28–30). The subjects' responses to the scale also depended on the scale and the subject population. In our study, the parent reports' scores were higher than child reports' scores in all subscales. The reason is perhaps that a portion of children in China are a little bit more ashamed to express their true thoughts to their parents. Good concordance between child reports and parent reports was observed for all subscales in our study. Children's age and cognitive development may pose some limitations in the completion of the questionnaire, so parents were required to complete the questionnaire, which will serve as references and supplements.

Indeed, there were some limitations in our present research. Although we included PBTs of all age groups and many kinds of cancers, we only conducted the study at one hospital, which cannot fully reflect the response of all Chinese children to this scale. Further multicenter collaborative studies are needed to expand the sample size in more representative hospitals.

## 5. Conclusion

To conclude, we developed the Chinese version of the PedsQL™ brain tumor module and verified its feasibility, reliability, and validity. This version was a first instrument used specifically to measure the HRQOL of PBTs in China. This module can be used to effectively monitor PBTs and conduct descriptive or exploratory studies to determine the risk factors affecting their HRQOL. We believe that wide application of Chinese version of the PedsQL™ brain tumor module provides an important method for formulating measures to improve patient prognosis and HRQOL. And this is also a new start for future multicenter researches in order to improve the HRQOL of PBTs in China.

## Data Availability

The original contributions presented in the study are included in the article/Supplementary Material, further inquiries can be directed to the corresponding author.
